# Design, Implementation and Validation of the Three-Wheel Holonomic Motion System of the Assistant Personal Robot (APR)

**DOI:** 10.3390/s16101658

**Published:** 2016-10-10

**Authors:** Javier Moreno, Eduard Clotet, Ruben Lupiañez, Marcel Tresanchez, Dani Martínez, Tomàs Pallejà, Jordi Casanovas, Jordi Palacín

**Affiliations:** 1 Department of Computer Science and Industrial Engineering, University of Lleida, 25001 Lleida, Spain; jmoreno@diei.udl.cat (J.M.); eclotet@diei.udl.cat (E.C.); robotica@udl.cat (R.L.); mtresanchez@diei.udl.cat (M.T.); dmartinez@diei.udl.cat (D.M.); jcasanovas@quimica.udl.cat (J.C.); 2 Barton Laboratory, Cornell University, Geneva, NY 14456, USA; tpc63@cornell.edu

**Keywords:** holonomic motion, assistant robot, mobile robot motion, omnidirectional wheel

## Abstract

This paper presents the design, implementation and validation of the three-wheel holonomic motion system of a mobile robot designed to operate in homes. The holonomic motion system is described in terms of mechanical design and electronic control. The paper analyzes the kinematics of the motion system and validates the estimation of the trajectory comparing the displacement estimated with the internal odometry of the motors and the displacement estimated with a SLAM procedure based on LIDAR information. Results obtained in different experiments have shown a difference on less than 30 mm between the position estimated with the SLAM and odometry, and a difference in the angular orientation of the mobile robot lower than 5° in absolute displacements up to 1000 mm.

## 1. Introduction

The uses of mobile robots are continuously increasing in non-industrial applications such as military and security settings [1], inspection of power lines in smart grids [2], crop-inspection in smart agriculture [3], disaster recovery [4], interaction with customers [5] and also helping people with mobility impairments [6]. Reports from diverse institutions such as the United Nations [7] and World Health Organizations [8] postulate that the proportion of people aged 60 or more will rise from 12% to 21% during the next 35 years as a result of a clear increase of human life expectancy and the development of assistant robots can be a technological tool that will contribute to increase the quality of life of elderly people and people with mobility impairments. In this direction, the combination of assistant mobile robots [9] and fixed domotic systems [10] can be used at home in an unstructured domestic environment and also contribute to supervise or develop some domestic tasks.

In this direction, the Assistant Personal Robot (APR) [9] proposed the conception of a new robotic assistant designed to operate in tight indoor spaces thanks to its holonomic motion system. The new contribution of this paper is the complete description of the optimized mechanical design, kinematics, and basic control of the three-wheel holonomic motion system implemented in the APR mobile robot. This complete description is proposed in order to foster replication and verification of the results. The paper ends with an empirical validation of the trajectory estimated by the internal control system which will be used in the future to improve the implementation of additional trajectory control procedures.

## 2. Background

Mobile robots can be classified accordingly to the motion system or by the type of mobility (Table 1). The motion system can be based on wheels, tracks, ball-shaped wheels or legs. The type of mobility can be classified as omnidirectional (or holonomic) or non-omnidirectional. The holonomic mobile robots have the advantage that they can change the direction of motion without having to perform intermediate rotation steps and they are able to move in all directions from a given starting point while simultaneously rotating [11].

Currently, in order to achieve mobility mobile robots are typically based on wheels. The use of wheels is more energy efficient than legged or treaded robots on hard and smoot surfaces [12,13,14]. The most popular wheeled mobile robots use two independent fixed driving wheels with two degrees-of-freedom (DOF) instead of three DOF (x,y,θ). These robots, like for example domestic cleaners, have only two actuators, requires less space to rotate around any point and this also allows three DOF, but their limitation is that they cannot perform holonomic motion such as sideways movements. An example of this type of mobile robot is shown on Table 1a, where this configuration is equipped with four fixed universal wheels.

To overcome this limitation, other mobile robots uses an omnidirectional motion system, like for example, mobile robots equipped with steerable and coordinated driving wheels and omnidirectional mobile robots based in wheels, ball-shaped wheel robots or legged robots. These devices offer interesting features when operating in tight spaces. Table 1b shows a mobile robot design equipped with steerable wheels published by Wada and Mori [15]. They allow both rotation and also sideways motion, but not simultaneously. This limitation can be overcome by using a holonomic omnidirectional motion system, which can move in all directions at any time without changing wheel direction, because they can achieve 3-DOF motion on a 2-dimensional plane and then the main limitation is wheel slippage [14].

Holonomic robots are based in the use of three or four omnidirectional wheels, see Table 1c, which are composed by several passive rollers or balls whose axes are tangent to the wheel circumference, and free to rotate. The three-wheeled omnidirectional mobile robots can have three independent actuators and they can achieve two independent translational and one rotational DOF, for the total of 3-DOF on a flat surface and maneuver and navigate in tight spaces such as in domestic environments. However due to their high center of gravity they have stability problems when they are moving on a ramp because of the triangular contact area with the ground [5,16]. This stability problem can be overcome by using four wheels with 4-DOF [11].

Table 1d shows a robot with ball-shaped wheels, such as the design proposed by West and Asada [17] that can run in any direction [18,19,20] but not over rough grounds or steps. Table 1 shows that omnidirectional mobile robots based in wheels can have universal or omnidirectional wheels. Alternatively, Table 1e shows also a legged robot because these can move in any direction and can move on any type of surface, however, the mechanism of legged robots are very complex and have velocity limitations [21].

In general, the principles of operation of omnidirectional mobile robots are based on kinematic models. One of the most popular references is the technical report by Muir and Neuman [12] which formulates the equations of motion of wheel-based mobile robots, incorporating also conventional omnidirectional and ball wheels. Currently there are a lot authors working in research about the locomotion of this mobile robots [5,13].

There are many holonomic mobile robot designs available in the literature. The first omnidirectional mobile robot was proposed in 1987 by Muir and Neuman [22] and was named Uranus. This proposal was based on introducing a methodology for the kinematic modeling of an omnidirectional wheeled mobile robot equipped with four omnidirectional wheels which was based in passive rollers arranged in an overlapping way. These wheels were positioned in pairs on the same axle but with opposite orientation. Alternatively, in 1996 Wada and Mori [15] proposed a new type of holonomic mobile robot which was equipped with steerable and coordinated driving wheels using conventional tires to provide an omnidirectional capability by actuating a wheels axis and a steering axis independently.

There are a several types of omnidirectional wheels but in all of them the principle of function is based in providing traction in the direction normal to the motor axis, and the use of inner passive rollers that can slide in the direction of the motor axis. These inner passive wheels, balls or rollers are placed along the periphery or the main wheels [14,23]. These omnidirectional wheels can be grouped in four types according to their traces.

**Figure 1:**
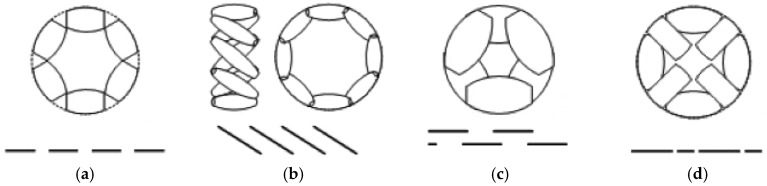
Types of omnidirectional wheels and their traces: (**a**) multiple passive rollers (or inner passive wheels) whose axes are positioned tangent to the main wheel circumference; (**b**) with the rollers arranged in an overlapping way where the contact between the wheels and the ground is continuous; (**c**) based on two overlapping parallel wheels; (**d**) based on using alternated passive rollers with different size and shape.

Figure 1a shows a wheel design which consists of multiple passive rollers (or inner passive wheels) whose axes are positioned tangent to the main wheel circumference. This construction cannot avoid the discontinuous traces and originate an irregular contact with surfaces because of gaps between successive rollers or wheels, which produce vibrations in the robot. To cancel these effects in these types of wheels there are some solutions which reduce the size gap between the passive rollers [24]. Mecanum [25] (Figure 1b), was invented in 1973 by Ilon, an engineer working for the Swedish Company Macanum AB and is other design type of wheel based on rollers arranged in an overlapping way in such a way that contact between the wheel and the ground is continuous. These wheels are thus usually positioned in pairs on the same axle but with opposite orientations to form a four-wheel structure. The drawback of these wheels is the generation of horizontal vibrations because of the parasite torques which are generated by the fact the contact point moves along a line parallel to the wheel shaft. The double wheel concept, presented in Figure 1c, is a solution based on two overlapping parallel wheels. The contact between the assembly wheel and the ground is continuous. This design generates an important horizontal vibration originated by the gaps between the rotating inner wheels. Finally, in the design of the Figure 1d, the contact points are in line, which avoids the horizontal vibrations and the alternated use of passive rollers of different sizes and shapes minimizes the gap between them thus causing little vertical vibration.

## 3. The Assistant Personal Robot

**Figure 2:**
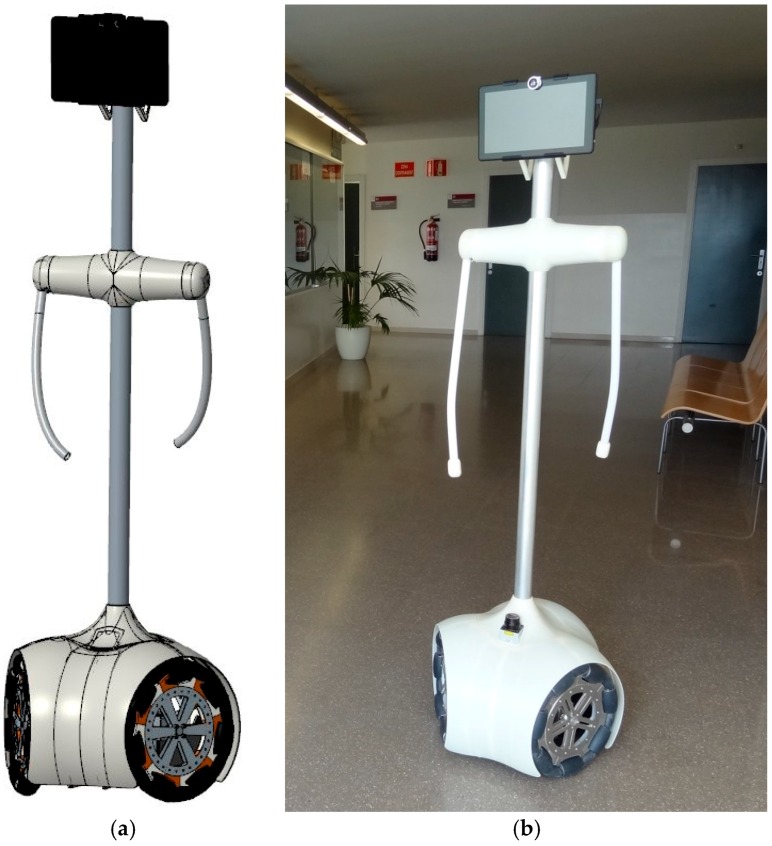
Assistant Personal Robot (APR): (**a**) CAD design; (**b**) prototype implementation.

The concept and design of the Assistant Personal Robot (APR, Figure 2) was presented in [9] with the aim to provide personal assistance services in households or institutions without interfering with the inhabitants. The APR was designed to be very maneuverable and capable of navigating in tight spaces. The physical design of the APR was inspired and includes several resemblances with humans in order to operate, maneuver and move the head and the arms in a similar way.

The APR (Figure 2) is a holonomic mobile robot, based on a mechanical structure where all elements of the APR are supported made with a combination of stainless steel and aluminum parts to guarantee the durability, resistance and control the weight. The structure of the APR is divided into a circular section base containing the motion system based on three omnidirectional wheels, and a thin body, which has two rotating arms and a multi-touch panoramic screen for interacting with humans. This agile, compact and reliable design avoids the presence of sharp edges or projecting parts, and facilitates its application in a domestic environment. Moreover, the mechanical structure formed by the body simplifies the application and development of supplementary mechanical devices.

The APR has a weight of 35 kg with the heavy elements placed on the base and close to the ground in order to provide a lower center of mass and stable displacement. The APR has a triangular contact area with the ground because of the three wheeled motion system. The base contains the motion system based on three omnidirectional wheels, the batteries, the LIDAR, and the main electronic boards. The external design of the base is completed with a bent plastic ABS case, which provides a flexible protection that will absorb part of the impact in case of collision.

The interactive zone contains a multi-touch panoramic screen and two shoulders with one degree of freedom in order to move the arms forwards and backwards. The chest and shoulders of the APR are located at approximately 1.3 m height which is slightly lower than the shoulders of an average human. This position was chosen in order to give elderly people direct access to hold the arms of the robot. The shoulders of the APR contain two heavy Micromotor DC motors which are connected to two soft arms with a 35 cm separation between them. The arms are 55 cm long just for esthetical reasons and can be used as a support by elder people when walking or used for basic gesture interactions. The arms are periodically moved during a forward displacement in order to mimic the natural movements performed by humans while walking.

The APR has a height of 164 cm and a width of 48 cm in order to simplify the remote tele-control of the mobile robot when passing through doorways, small corridors or complicated paths. The most characteristic parts of the APR is the holonomic motion system and this paper describes the mechanical implementation and the control of the motion system.

### 3.1. Mechanical Implementation of the Motion System of the APR

**Figure 3:**
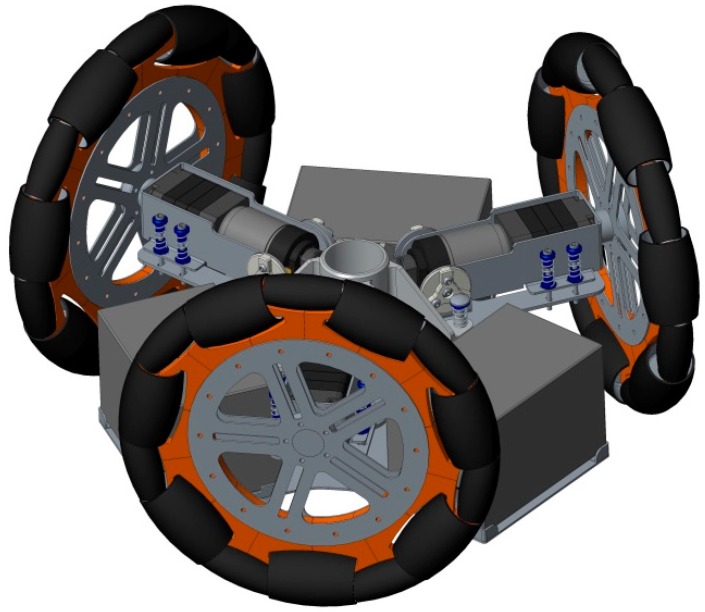
CAD model of the motion system based on the use of three omnidirectional wheels shifted 120°.

The omnidirectional robots can maneuver in small spaces and perform complex trajectory paths. The inclusion of a circular design also minimizes the probability of it getting accidentally caught on furniture objects such as mats, curtains or clothing. Figure 3 shows the motion system of the APR, which is based in the use of three omnidirectional wheels, shifted 120° and composed of passive rollers. This three-wheeled robot has three independent geared DC motors attached to the wheels and they can achieve 3-DOF. Each wheel has the same distance, *R*, from its center to the center of the mobile robot.

In the future, this motion system will include additional suspension in order to minimize the vertical vibrations caused by the gap between the omnidirectional wheels. The inclusion of a suspension system based on springs allows the adaptation of the preload to different floor conditions.

The design of the APR is based on the operating principle of a pendulum. This design allows individual damping and pillar oscillation. The pivoting point is placed over the center of mass, mainly formed by the batteries, so the forces that appear during the oscillations tend to put the body to rest, reaching the natural damping equilibrium of the device.

### 3.2. Omnidirectional Wheels

**Figure 4:**
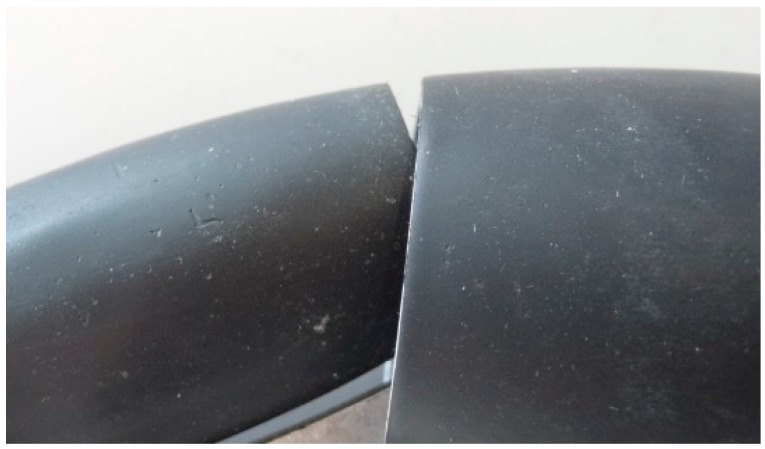
Detail of the gap between the passive rollers.

The design of the omnidirectional wheels is based on alternating passive rollers with different size and shapes in order to minimize the gap between the rollers (see Figure 4) which causes vertical vibration. This implementation allows wheel spin and perpendicular displacements from the wheel forwarding direction and thus the direct displacement in any direction.

**Figure 5:**
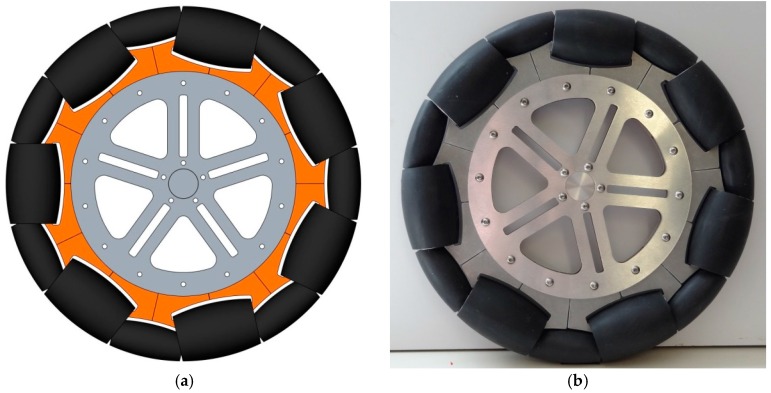
Design of the omnidirectional wheel: (**a**) CAD model (**b**) prototype implementation.

**Figure 6:**
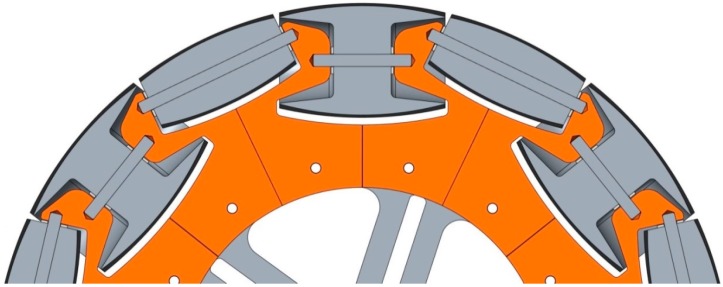
CAD section showing the alternate use of the two passive roller types.

The omnidirectional wheel used in the APR has seven passive rollers of two different types, whose axes are positioned tangent to the main wheel circumference. Figure 5a shows the CAD design of the omnidirectional wheels and Figure 5b the prototype implementation. Additionally, Figure 6 shows a detail of the passive rollers and the shape of the circumference of the wheel.

**Figure 7:**
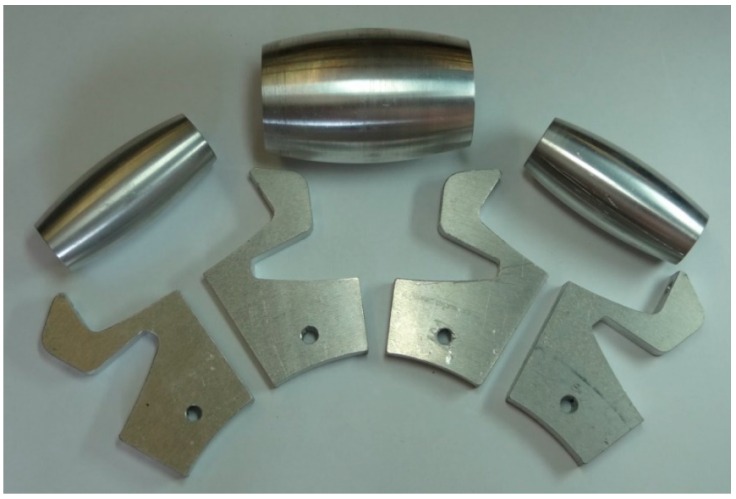
Exploded view of the basic components of the wheel.

**Figure 8:**
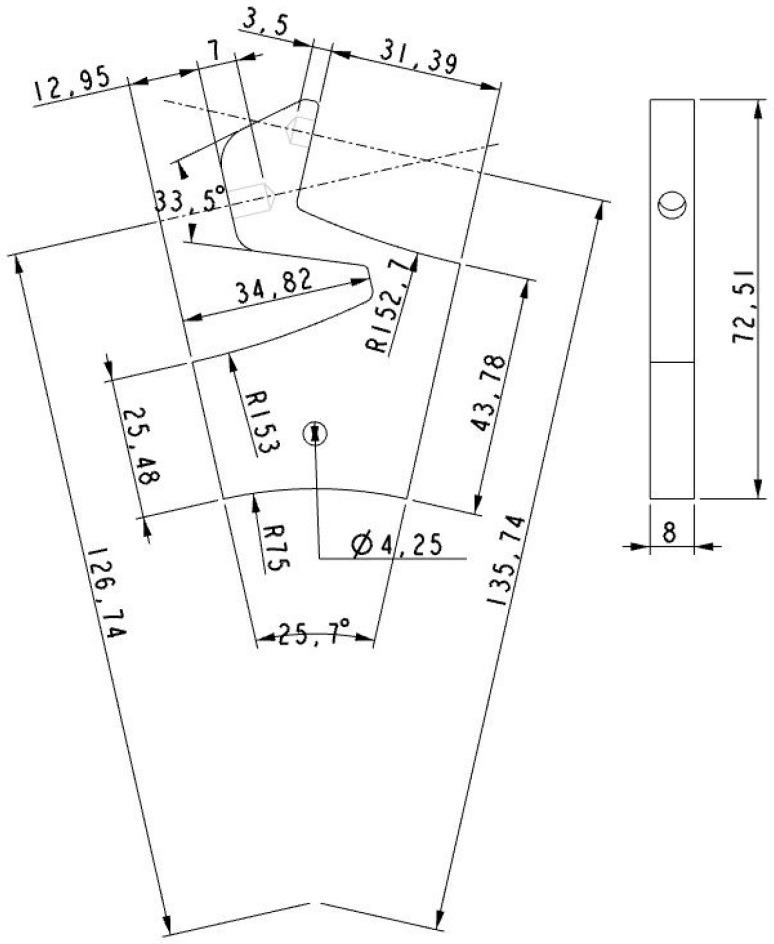
Drawing front and side elevation of roller bracket (units in mm).

**Figure 9:**
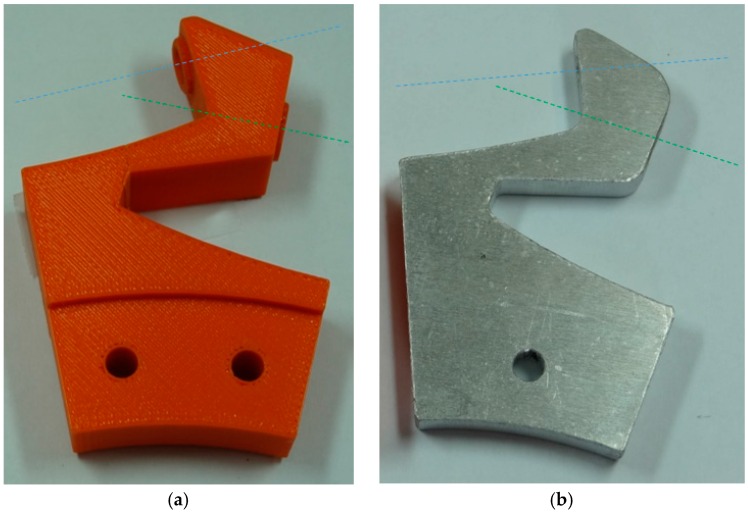
Rollers brackets with axis of big passive roller (green axis) and small passive roller (blue axis). (**a**) Example made of ABS plastic with a rapid prototyping 3D printer; (**b**) made of aluminum with laser cut and numeric control.

The shape of bracket rollers allows that the small roller can be partially housed in the big roller, allowing dismiss the gap between the rollers. The circumference of the wheels has an external diameter of 300 mm and a width of 46 mm. The weight of each omnidirectional wheel implemented in aluminum is 2.6 kg. The main parts of the omnidirectional wheel (Figure 7) are the roller brackets (Figure 8 and Figure 9) and the passive rollers. The shape of the bracket rollers allows the overlapping of the rollers in order to reduce the size of the gap (2.5 mm in this implementation) and to minimize the vertical vibrations in the transitions. Figure 7 shows an exploded view of the basic components of the wheel.

In the first prototype some parts like rollers and rollers brackets were made in ABS plastic using the technique of rapid prototyping by fusion deposition modeling (FDM). The main advantage of a rapid prototyping manufacturing is the simple implementation of any type of complex piece. Nevertheless, several plastic rollers brackets end up breaking in areas of accumulation of tension due to fatigue stresses.

Figure 8 shows the simplified symmetric aluminum implementation of the roller brackets in order to improve the durability, reduce the cost and simplify the construction of the wheels. The rollers brackets are simple part which is based on an aluminum plate (8 mm height), cut with a laser by numeric control and combined with holes also implemented with a computer numeric control to allow the correct orientation of edges to passive rollers (see Figure 9).

**Figure 10:**
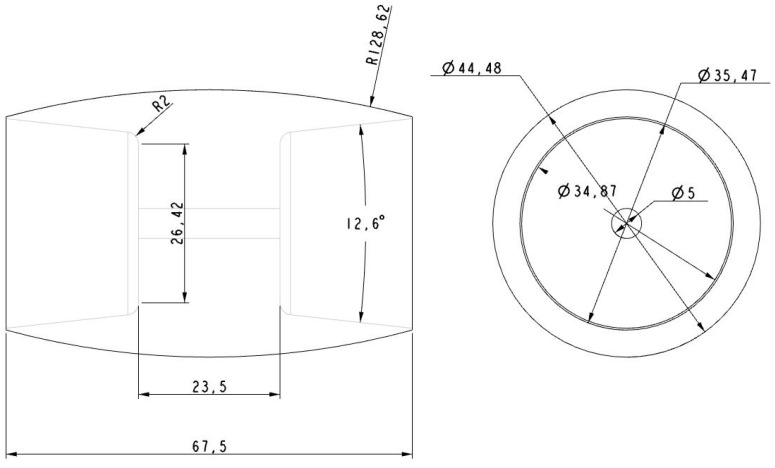
Drawing front and side elevation of big passive roller (units in mm).

**Figure 11:**
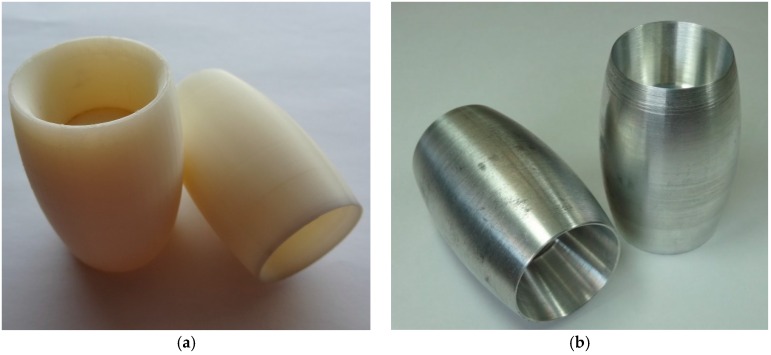
Big passive roller implemented in (**a**) plastic ABS and (**b**) aluminum.

**Figure 12:**
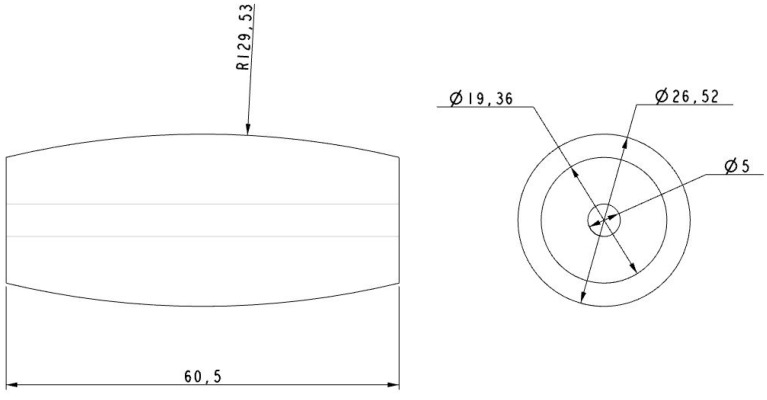
Drawing front and side elevation of small passive roller (units in mm).

**Figure 13:**
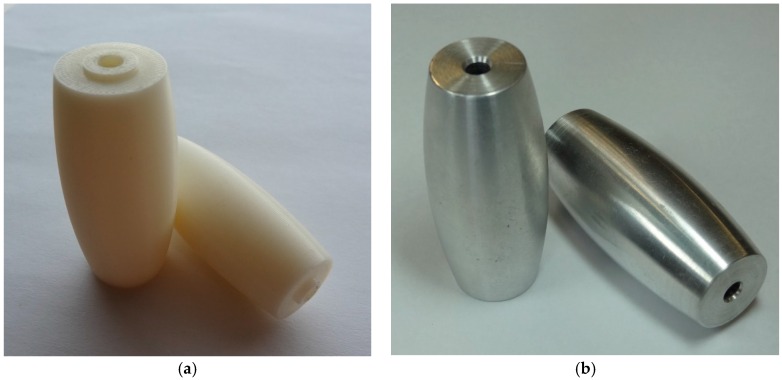
Small passive roller implemented in (**a**) plastic ABS and (**b**) aluminum.

The rollers were also made of aluminum using a numerical control lathe (see Figure 10, Figure 11, Figure 12 and Figure 13) and covered with 0.8 mm adherent plastic to increment the grip. The rollers with barrel-shaped are alternated in order to achieve a continuous contact. The big passive roller has a maximum diameter of 44.48 mm and a length of 67.5 mm (see Figure 10 and Figure 11). The small passive roller has a maximum diameter of 26.52 mm and a length of 60.5 mm (see Figure 12 and Figure 13).

### 3.3. Inverse Kinematic Model

The functioning principle of the three omnidirectional wheels shifted 120° is based on providing traction in the direction normal to the motor axis while the passive rollers slide in the direction of the motor axis. The design of the omnidirectional motion allows simultaneous sideways and rotation motion. However, for the shake of simplicity, the study the mobile robot mobility has been divided in two parts: translation and rotation. The relationship between the forces exerted by the wheels and the robot movement was based in a dynamic model. The linear and angular velocities of the mobile robot are the inputs of kinematic model whereas the outputs are robot wheels velocities.

**Figure 14:**
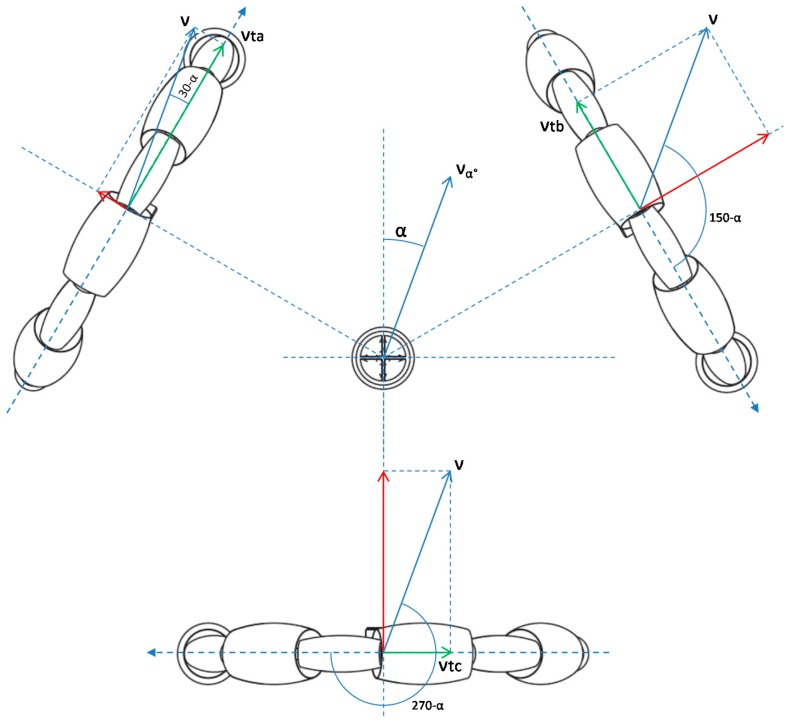
Kinematic diagram of the translation of the mobile robot: v and α define the velocity vector of the mobile robot in polar form and $v_{ta}$, $v_{tb}$, and $v_{tc}$ are the velocity in each of the wheels caused by translation.

Figure 14 show the input velocity vector of the mobile robot, $\vec{v}$, which can be represented in polar form as (υ, α). This velocity vector is the target vector which is discomposed in each wheel in two vectors: one projection is normal direction to the motor axis and the other is transversal. The translation velocities of the wheels a, b, c have been named as $v_{ta}$, $v_{tb}$, and $v_{tc}$. The wheels can slide in the direction of the motor axis thanks to the use of passive rollers.

**Figure 15:**
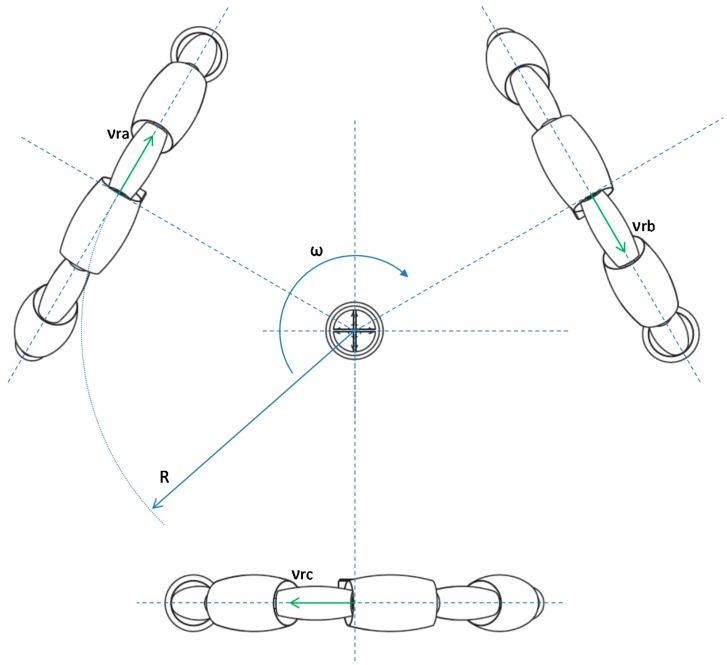
Kinematic diagram of the rotation of the mobile robot: ω is the rotation and $v_{ra}$, $v_{rb}$, and $v_{rc}$ are the velocity in each of the wheels caused by rotation motion. R is the distance between the wheels and the center of mobile robot.

Figure 15 shows the projection on the normal direction, $v_{ra}$, $v_{rb}$, and $v_{rc}$ due to the rotation ω. The rotational movement provides equivalent speed at each wheel.

The equations that describe the velocity of each wheel, $\nu_{w}$, in terms of rotation and translation are Equations (1) and (2):

(1) $$\nu_{w}\left(\nu_{a},\nu_{b},\nu_{c}\right)=v_{traslation}\left(\nu_{ta},\nu_{tb},\nu_{tc}\right)+v_{rotation}\left(\nu_{ra},\nu_{rb},\nu_{rc}\right),$$

(2) $$\nu_{w}\left(\nu_{a},\nu_{b},\nu_{c}\right)=v\cdot \left(\begin{array}{c} \cos\left(30-\alpha \right)\\ \cos\left(150-\alpha \right)\\ \cos\left(270-\alpha \right) \end{array}\right)+\omega \cdot R,$$

where $\nu_{w}\left(\nu_{a},\nu_{b},\nu_{c}\right)$ are the velocity of the wheels, v is the module of the mobile robot velocity, α is the angular orientation expressed in degrees, ω is the rotation of the mobile robot and R is the distance between the wheels and the center of mobile robot (220 mm). Equations (1) and (2) can be used to estimate the velocity of the wheels required to move the mobile robot at a desired velocity and angular rotation.

### 3.4. Kinematic Model

**Figure 16:**
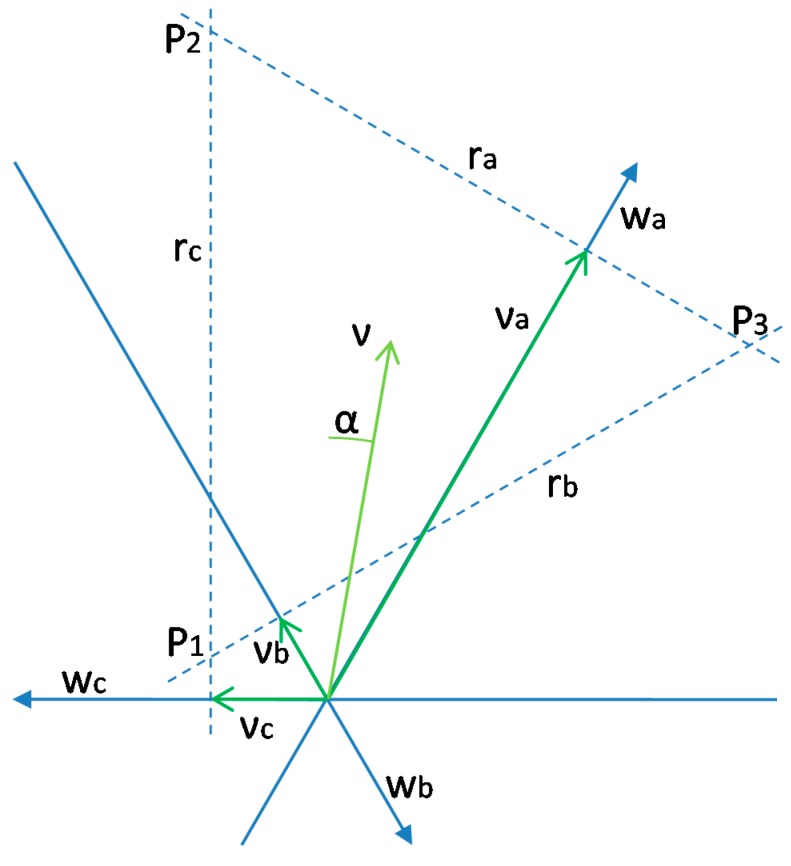
Graphic diagram to solve the kinematic of mobile robot based on use the projections of the velocity vectors of each wheel.

The kinematic model of the mobile robot is based on the analysis of the rotation of the wheels in order to estimate υ (in m/s), α (in rad) and ω (in rad/s) of the mobile robot. The APR estimates the velocity of the wheels by using the encoders in the motors of the wheels. The computation of the kinematic model from Equation (2) is not trivial because it is a non-linear system. In this paper, the kinematic model of the mobile robot has been obtained graphically because only the velocities of the wheels are available. Figure 16 shows the kinematic diagram of the motion of the mobile robot, where $\nu_{a},\nu_{b},\nu_{c}$ are the velocity of the wheels, represented on the projections of the directions of the wheels; $w_{a}$, $w_{b}$, $w_{c}$,$\;r_{a}$, $r_{b}$, $r_{c}$ are the projections of the possible velocity vector of motion; and $P_{1}$, $P_{2}$, $P_{3}$ are the points cuts. The interpretation of Figure 16 is as follows: the dashed lines show the normal direction to the motor axis of each wheel and the arrow indicates the positive direction of rotation of the wheel and on which we project the speed of each wheel, which it is a sum of the speed of translation and rotation. In the case of no rotation in the displacement of the mobile robot (Figure 14), the velocity vector of translation are at some point at the normal line established at the end of the vector speed of each wheel, and then the solution is the cutoff point. In the case of rotation in the displacement of the mobile robot (Figure 15), the velocity vector is equidistance to the normal lines because the rotational movement provides equivalent speeds at each wheel.

The equations to calculate the projection line of velocity of each wheel $r_{a}$, $r_{b}$ $r_{c}$, are:

(3) $$r_{a}:y=-\tan30\cdot x+\left(v_{a}\cdot \cos30+v_{a}\cdot \tan30\cdot \sin30\right)=-\frac{\sqrt[{2}]{3}}{3}\cdot x+v_{a}\left(\frac{\sqrt[{2}]{3}}{2}+\frac{\sqrt[{2}]{3}}{3}\cdot \frac{1}{2}\right)\;r_{a}:y=-\frac{\sqrt[{2}]{3}}{3}\cdot x+\frac{2\sqrt[{2}]{3}}{3}v_{a}$$

(4) $$r_{b}:y=\tan30\cdot x+\left(\left(-v_{b}\right)\cdot \cos30+\left(-v_{b}\right)\cdot \tan30\cdot \sin30\right)=\frac{\sqrt[{2}]{3}}{3}\cdot x-v_{b}\left(\frac{\sqrt[{2}]{3}}{2}+\frac{\sqrt[{2}]{3}}{3}\cdot \frac{1}{2}\right)\;r_{b}:y=\frac{\sqrt[{2}]{3}}{3}\cdot x-\frac{2\sqrt[{2}]{3}}{3}v_{b}$$

(5) $$r_{c}:x=-v_{c}$$

This equidistant point can be obtained as the centroid of the triangle formed, which can be obtained using the cutoffs of $r_{a}$, $r_{b}$, $r_{c}$:

(6) $$r_{c}\Leftrightarrow r_{b}:P_{1}=\left(-v_{c},-\frac{\sqrt[{2}]{3}}{3}\left(v_{c}+2v_{b}\right)\right)$$

(7) $$r_{c}\Leftrightarrow r_{a}:P_{2}=\left(-v_{c},\frac{\sqrt[{2}]{3}}{3}\left(v_{c}+2v_{a}\right)\right)$$

(8) $$r_{a}\Leftrightarrow r_{b}:P_{3}=\left(v_{a}+v_{b},\frac{\sqrt[{2}]{3}}{3}\left(v_{a}-v_{b}\right)\right)\;G=\left(\frac{P_{1x}+P_{2x}+P_{3x}}{3},\frac{P_{1y}+P_{2y}+P_{3y}}{3}\right)=\left(\frac{v_{a}+v_{b}-2v_{c}}{3},\frac{\sqrt[{2}]{3}\left(v_{a}-v_{b}\right)}{3}\right)$$

And then, the equations for calculating the velocity vector of the mobile robot, $\vec{v}$, which can be represented in polar form as (υ, α) and ω are:

(9) $$v=\sqrt[{2}]{{\left(G_{x}\right)}^{2}+{\left(G_{y}\right)}^{2}}=\sqrt[{2}]{{\left(\frac{v_{a}+v_{b}-2v_{c}}{3}\right)}^{2}+\frac{{\left(v_{a}-v_{b}\right)}^{2}}{3}}$$

(10) $$\alpha =90-\tan^{-1}\left(\frac{\sqrt[{2}]{3}\left(v_{a}-v_{b}\right)}{v_{a}+v_{b}-2v_{c}}\right)$$

(11) $$\omega \cdot R=G_{x}-r_{c}=\frac{v_{a}+v_{b}-2v_{c}}{3}-\left(-v_{c}\right)\;\omega =\frac{v_{a}+v_{b}+v_{c}}{3R}$$

being v the velocity of the mobile robot, α the angular orientation and ω the angular rotation of the mobile robot.

## 4. Control of the Motion System

**Figure 17:**
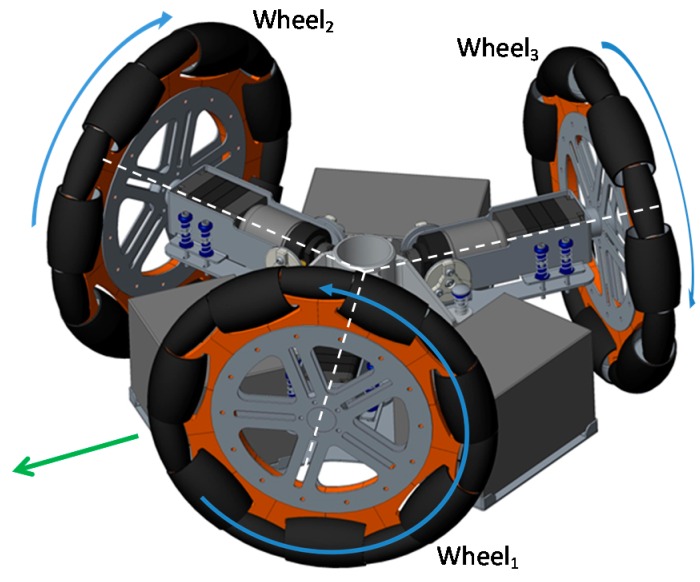
Detail of the labeling and positive angular velocity of the wheels. The green arrow depicts the front of the mobile robot.

The motion system of the APR is controlled with an ARM Cortex-M4 microcontroller [9]. The control of the motion is based on the information provided by the encoders of the DC motors of the mobile robot. The magnetic encoders available in the low cost DC motors generate three impulses per turn and the motor velocity is estimated by counting the time between the pulses with a 16 bits timer counter so there are tree velocity estimates per motor turn. The wheel velocity is directly estimated by dividing the motor velocity by the fixed mechanical gear ratio of the DC motors (43:1). The microcontroller generates three Pulse Width Modulation (PWM) signals which are expressed in percentage (%) and applied to three H-bridges in order to convert the 12 V (13.8 V at full charge) of the battery into an average DC voltage in the motors. Figure 17 shows the wheel labeling used: wheel 1 is the front-left, wheel 2 is the front-right, and wheel 3 is the back wheel.

**Figure 18:**
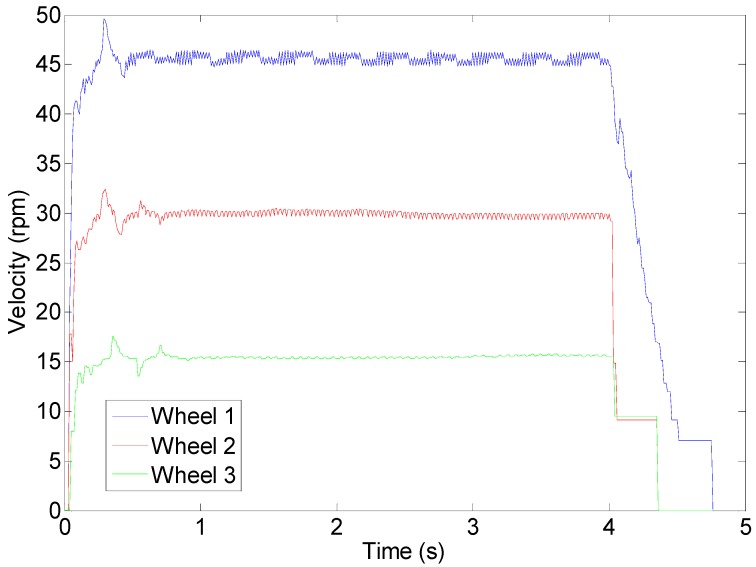
Wheel velocity profile during an open loop operation.

**Figure 19:**
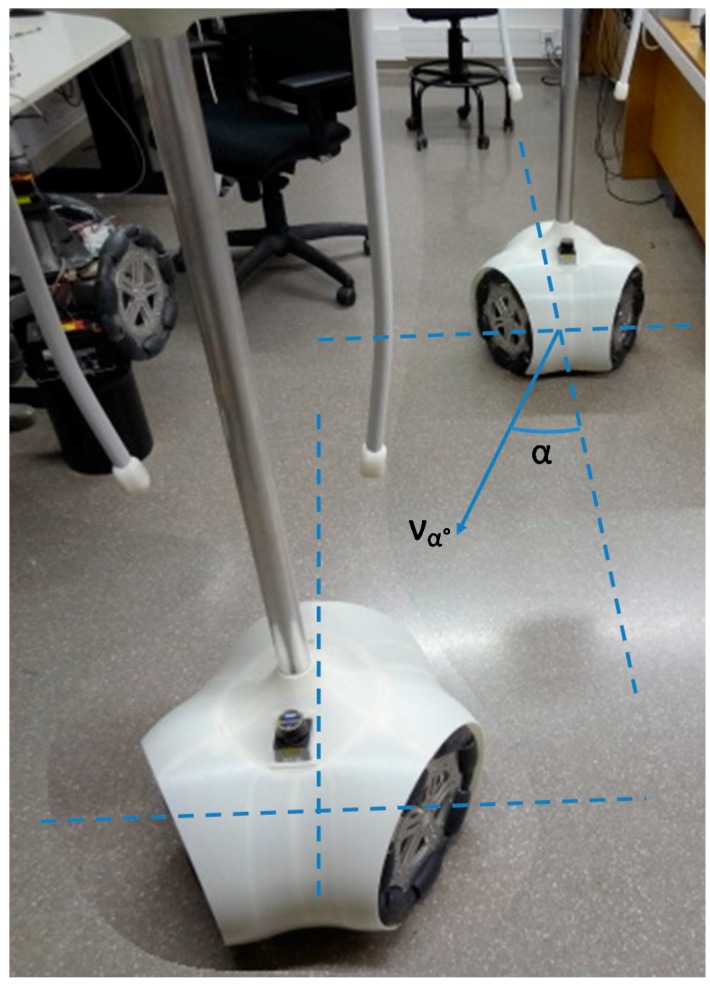
Composite image created to show the relative displacement of the mobile robot originated by wheel velocity profile of Figure 18. The APR maintains the absolute angular orientation.

The DC motors are powerful enough to displace the 35 kg of the mobile robot with a forward velocity comparable with that of a walking human. Figure 18 shows the profile of the velocity of the wheels of the APR in case of applying a fixed PWM of 62%, 40%, 25% during 4 s to the motors of the APR in an open loop operation without any feedback control. The average velocities of the wheels were approximately 45, 30 and 15 rpm respectively. Figure 19 shows an image of the initial and ending mobile robot position obtained after this experiment. Figure 18 shows that, in this case, the wheels reach the 80% of the maximum speed in only 0.1 s which means that the mobile robot reaction is very fast but then the mechanical stress suffered by the onboard mechanical and electronic elements is also very high. Figure 18 also shows that there are an unwanted peak prior velocity stabilization. Despite these cited effects, the trajectory of the APR in the case of using an open loop in the motors is very stable and visually predictable. The only drawback is a violent acceleration and deceleration due to the power of the motors used in the motion system.

**Figure 20:**
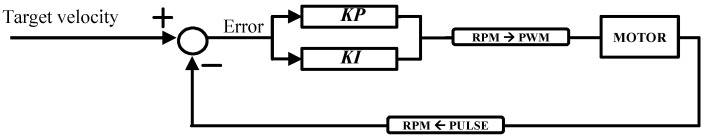
Detail of the PI controller implementation.

The control of the velocity of the wheels of the mobile robot has been performed by applying a conventional Proportional, Integral, and Derivative (PID) controller to the velocity of the DC motors in a closed loop control. In general, the PID controller is designed to provide fast reaction to target changes. However, an adequate design of the PID also allows a smoothed supervised evolution of the power applied to the device controlled. In this paper, the tuning of the PID controller constants has been performed by a trial and error procedure with the aim of obtaining a smooth, stable and visually predictable mobile robot motion. At the end of this subjective manual tuning procedure the PID controller has been simplified as a Proportional and Integral (PI) controller with KP and KI values of 0.01 and 1.50 respectively (Figure 20). As a summary, the control procedure applied is as follows: (1) a target velocity (in rpm) is defined for a wheel; (2) one internal timer is used to count the encoder pulses and to estimate the velocity of the DC motor velocity (in rpm); (3) the motor velocity is then converted to wheel velocity (in rpm) by applying the gear ratio; (4) the target and measured wheel velocity are then compared; and (5) the difference is multiplied by KD and KI and integrated (cumulated). In this paper, the cumulative difference expressed in rpm is converted in PWM percentage by applying a conversion factor of approximately 1.42%/rpm.

**Figure 21:**
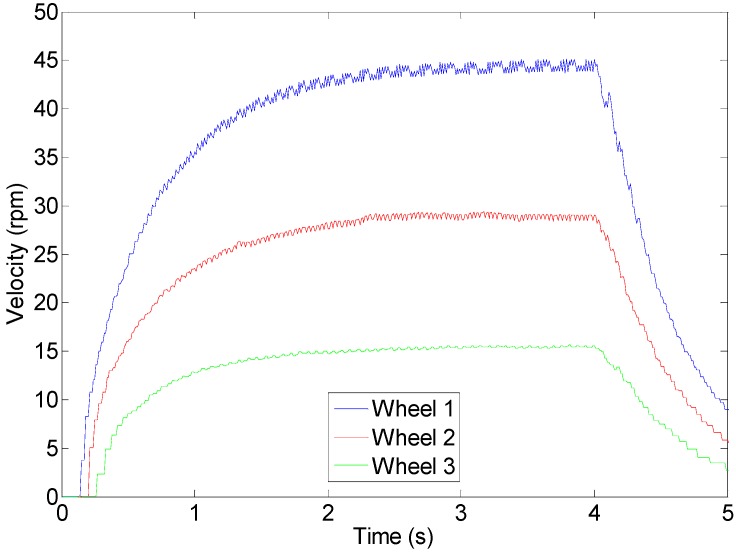
Wheel velocity profile during a closed loop operation.

Figure 21 shows the profile of the velocity of the wheels of the APR in case of activating the DC motors during 4 s and controlling the velocity with the proposed closed loop. Figure 21 shows that, in this case, the wheels reach the 80% of the maximum speed in more than 1 s. The drawback of this closed loop control is that the integral controller does not stop the mobile robot instantaneously because the deceleration profile is similar to the acceleration profile. In the practice, the selection of a target wheel velocity up to the 95% of the maximum velocity of the motors creates a smooth acceleration and deceleration profile that not saturate the motors, reduce vibrations and improve the external predictability of the trajectory of the mobile robot. In any case, the high order control system of the APR can force an instantaneous stop of the mobile robot by disconnecting the integral controller and short-cutting the H-bridges in order to block the motors (applying an electrical brake).

**Figure 22:**
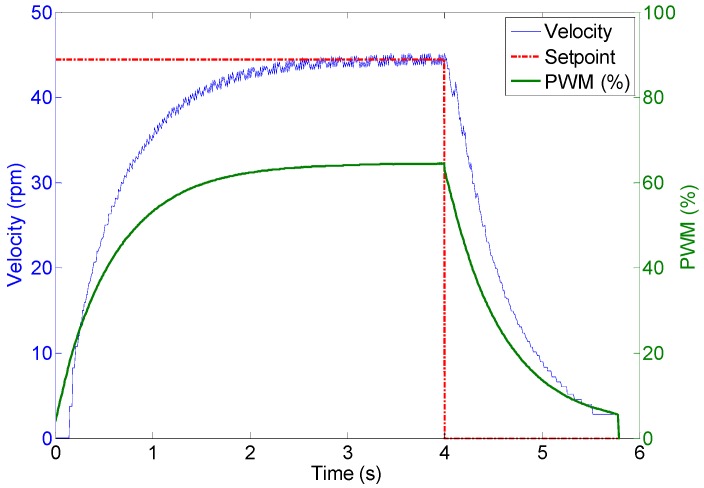
Wheel 1. Evolution of wheel velocity and applied motor PWM for a target velocity of 44 rpm.

**Figure 23:**
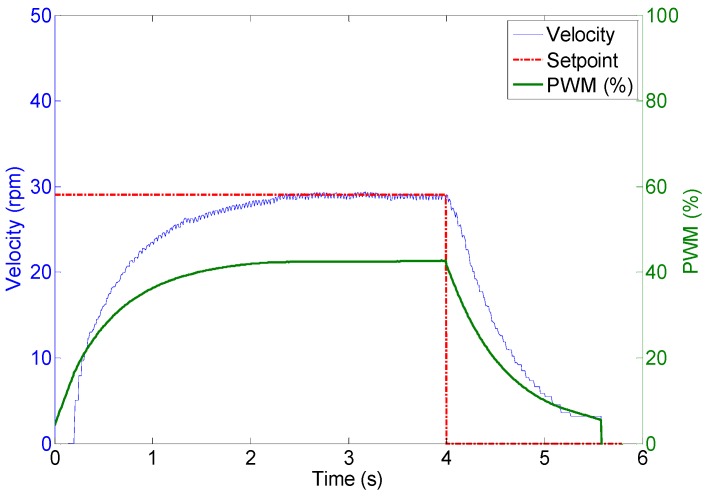
Wheel 2. Evolution of wheel velocity and applied motor PWM for a target velocity of 29 rpm.

**Figure 24:**
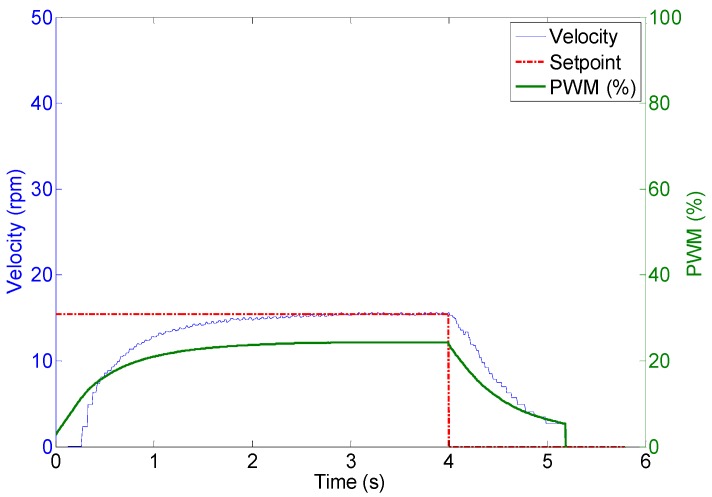
Wheel 3. Evolution of wheel velocity and applied motor PWM for a target velocity of 15 rpm.

Finally, Figure 22, Figure 23 and Figure 24 show the target velocity setpoint applied to the PID of the wheels, the evolution of the wheel velocity estimated with the encoders, and the evolution of the PWM applied to the DC motors. This control system is simple but extremely effective. For example, when the APR is remotely tele-operated this control system generates always predictable trajectories which are very easy to supervise. The combination of the inverse kinematic model and the control system takes full advantage of the holonomic motion of the mobile robot even in case of severe trajectory changes.

## 5. Validation

The validation of the three-wheels holonomic motion system consisted of the comparison of the trajectory of the mobile robot obtained by using two alternative methods: (1) by using a SLAM procedure (used in [26]) for precise absolute positioning based on the information provided by the onboard LIDAR; and (2) by using the information of the encoders and the kinematic model presented in this paper. The main objective of the validation procedure is to compare both measurements.

**Figure 25:**
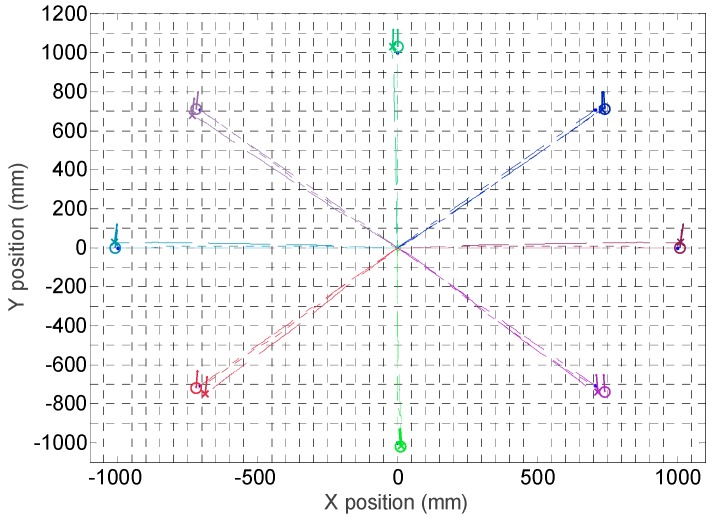
Trajectories followed by the mobile robot in eight displacements. The circle depicts the final position and orientation of the mobile robot obtained with the SLAM procedure. The cross depicts the final position and orientation of the mobile robot according the information of the encoders and the kinematic model.

Figure 25 summarizes eight trajectories of the mobile robot obtained in a first validation experiments. In this case, the mobile robot has to maintain its orientation while moving a straight fixed distance in a predefined angular direction, α (see Figure 19 for a reference). This capability of moving without changing the orientation is a characteristic feature of a holonomic motion system that is validated in this experiment. The angular direction tested in the displacements were (0°, 45°, 90°, 135°, 180°, 225°, 270°, 315°) and the angular velocity was always 0°.

The procedure of each measurement is as follows: (1) a specific mobile robot velocity, distance and angular direction of the displacement is fixed; (2) the mobile robot computes the angular velocity of the wheels according the inverse kinematic model, this velocity will remain constant during the validation experiment; (3) the mobile robot estimates the ideal-time required for the displacement according the kinematic model; (4) the internal time measurement is reset; (5) the angular velocities of the wheels are stablished as setpoints of the three PI controllers of the mobile robot and the motion is automatically started; (6) when reaching the ideal-time of the experiment, the setpoints of the angular velocities of the wheels are set to zero and the PI controllers deaccelerate the mobile robot until completely stopping the device. Figure 25 summarizes the mobile robot trajectories obtained with the information of the encoders and the kinematic model and with the SLAM procedure: the circle depicts the position according the SLAM procedure and the cross according the information of the encoders; in both cases a small line also depicts the final orientation of the mobile robot which is approximately the same in all trajectories.

**Figure 26:**
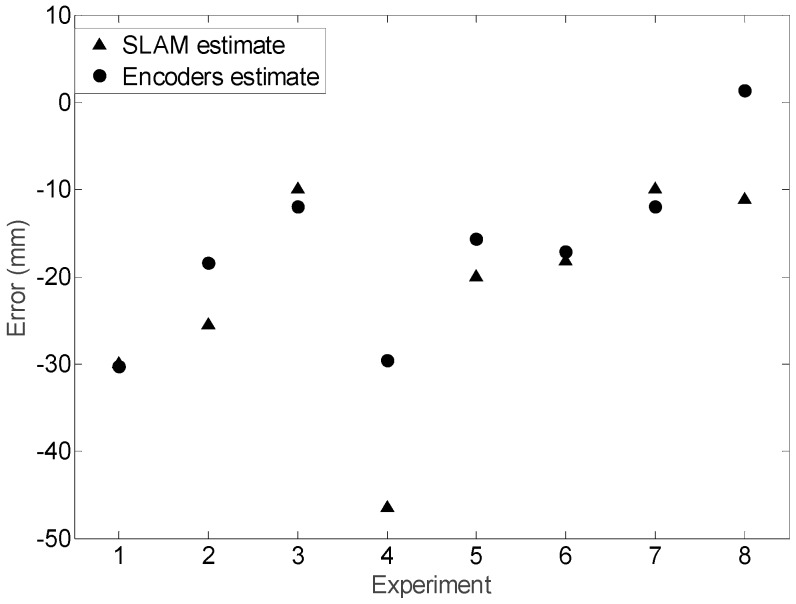
Absolute displacement error obtained when comparing the planned displacement and the final displacement estimated with the SLAM procedure and with the encoders and the kinematic model.

**Figure 27:**
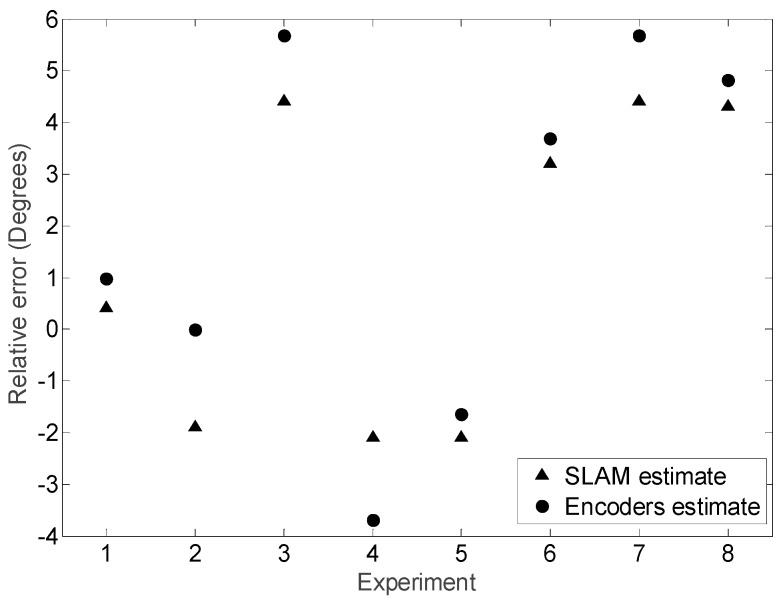
Absolute final angular orientation error obtained when comparing the planned angular orientation and the final orientation estimated with the SLAM procedure and with the encoders and the kinematic model.

The results shown in Figure 25 show small differences in the mobile robot trajectory estimated with the SLAM procedure and with the encoders and kinematic model of the motion system. Figure 26 details the absolute error in the position estimated with the LIDAR and the kinematic model compared with the planned displacement. The maximum difference between the desired theoretical and estimated distance was always lower than 60 mm in all measurements corresponding to a planned straight trajectory displacement of 1000 mm with the mobile robot. Moreover, the difference of the position estimated with the SLAM procedure and the kinematic model were always lower than 30 mm, which may be considered a very low difference. Finally, Figure 27 shows the differences between the planned angular orientation of the mobile robot and the values estimated according the SLAM procedure and according the information of the encoders and the kinematic model which are always lower than 5° in all trajectories measured in this first validation experiment. The displacement estimated with the information of the encoders has been also compared with manual measurements and with measurements obtained with an external LIDAR [27] obtaining similar results than the displacement estimated with the SLAM procedure. However, the use of the SLAM onboard procedure has the advantage of an easy synchronization between the trajectories estimated with both methods.

**Figure 28:**
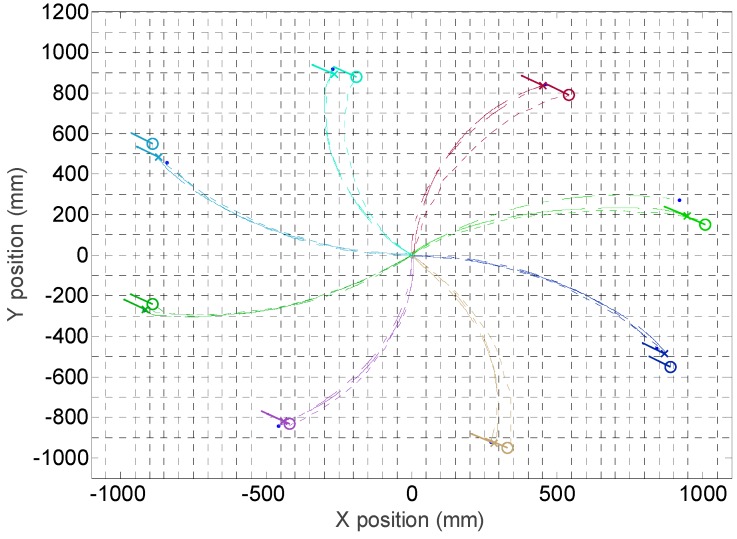
Trajectories followed by the mobile robot in eight displacements. The circle depicts the final position and orientation of the mobile robot obtained with the SLAM procedure. The cross depicts the final position and orientation of the mobile robot according the information of the encoders and the kinematic model.

Finally, Figure 28 summarizes the mobile robot trajectories obtained with the information of the encoders and the kinematic models and with the SLAM procedure. In this second validation experiment, the mobile robot has to maintain its orientation while moving a fixed distance following a curved path. The angular direction tested in the displacements were (0°, 45°, 90°, 135°, 180°, 225°, 270°, 315°) and the angular velocity was always 5% of the maximum velocity. In these measurements, the difference between the position estimated with the SLAM procedure and with the encoders and the kinematic model was again lower than 30 mm in a total distance of 1000 mm whereas the difference between the estimates of the angular orientation of the mobile robot was also lower than 5°.

The comparative empirical results obtained validate the design of the three-wheel holonomic motion system for mobile robot displacement and the utility of the proposed inverse kinematic model in order to estimate the relative displacement of the mobile robot according the information of the encoders of the wheels.

## 6. Conclusions

This paper presents the design and implementation of the mechanical design of the three-wheel holonomic motion system implemented in the Assistant Personal Robot (APR), a mobile robot designed to operate at home. The paper analyzes the inverse and direct kinematics of the motion, describes the control system and shows the result of different experiments proposed to validate the complete motion system. The trajectory of the mobile robot has been estimated by using the information of the encoders of the wheels and the proposed kinematic model. This trajectory has been compared with the trajectory obtained with a SLAM procedure based on the information obtained by an onboard LIDAR. Results have shown a discrepancy in both estimates of less than 30 mm in distance, and less than 5° in angular orientation for absolute displacements of up to 1000 mm. These results confirm the utility of the three-wheel holonomic motion system proposed for the implementation of an Assistant Personal Robot.

## Figures and Tables

**Table 1:** Classification of mobile robots based on the motion system and type of mobility.

Motion System Based on				
Wheels			Ball	Legs
Universal		Omnidirectional		
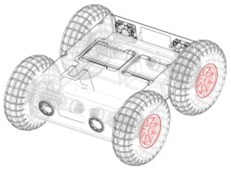	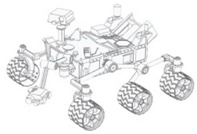	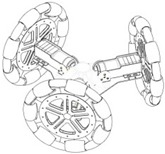	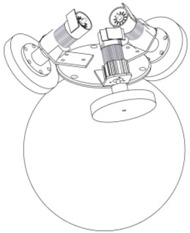	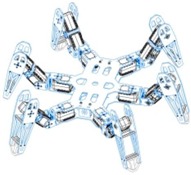
		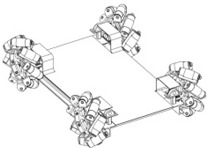		
(**a**)	(**b**)	(**c**)	(**d**)	(**e**)
Not omnidirectional	Omnidirectional			
Type of mobility				
